# Two-Stage Fermentation of *Lipomyces starkeyi* for Production of Microbial Lipids and Biodiesel

**DOI:** 10.3390/microorganisms9081724

**Published:** 2021-08-13

**Authors:** Le Zhang, Ee Yang Lim, Kai-Chee Loh, Yanjun Dai, Yen Wah Tong

**Affiliations:** 1 NUS Environmental Research Institute, National University of Singapore, 1 Create Way, Create Tower #15-02, Singapore 138602, Singapore; erizle@nus.edu.sg (L.Z.); chelohkc@nus.edu.sg (K.-C.L.); 2 Energy and Environmental Sustainability for Megacities (E2S2) Phase II, Campus for Research Excellence and Technological Enterprise (CREATE), 1 Create Way, Singapore 138602, Singapore; ee_yang@u.nus.edu (E.Y.L.); yjdai@sjtu.edu.cn (Y.D.); 3 Department of Chemical and Biomolecular Engineering, National University of Singapore, 4 Engineering Drive 4, Singapore 117585, Singapore; 4 School of Mechanical Engineering, Shanghai Jiao Tong University, Shanghai 200240, China

**Keywords:** two-stage fermentation, microbial lipids, biodiesel, oleaginous yeast, waste-to-resource, orange peel hydrolysate

## Abstract

The high operating cost is currently a limitation to industrialize microbial lipids production by the yeast *Lipomyces starkeyi*. To explore economic fermentation technology, the two-stage fermentation of *Lipomyces starkeyi* using yeast extract peptone dextrose (YPD) medium, orange peel (OP) hydrolysate medium, and their mixed medium were investigated for seven days by monitoring OD_600_ values, pH values, cell growth status, C/N ratios, total carbon concentration, total nitrogen concentration, residual sugar concentration, lipid content, lipid titer, and fatty acids profiles of lipids. The results showed that two-stage fermentation with YPD and 50% YPD + 50% OP medium contributed to lipid accumulation, leading to larger internal lipid droplets in the yeast cells. However, the cells in pure OP hydrolysate grew abnormally, showing skinny and angular shapes. Compared to the one-stage fermentation, the two-stage fermentation enhanced lipid contents by 18.5%, 27.1%, and 21.4% in the flasks with YPD medium, OP medium, and 50%YPD + 50%OP medium, and enhanced the lipid titer by 77.8%, 13.6%, and 63.0%, respectively. The microbial lipids obtained from both one-stage and two-stage fermentation showed no significant difference in fatty acid compositions, which were mainly dominated by palmitic acid (33.36–38.43%) and oleic acid (46.6–48.12%). Hence, a mixture of commercial medium and lignocellulosic biomass hydrolysate could be a promising option to balance the operating cost and lipid production.

## 1. Introduction

Imminent energy shortages, organic wastes management, and the environmental impacts of fossil fuels are the dilemmas we face currently [1]. To mitigate these issues, many renewable energy technologies (e.g., microbial fermentation, gasification, and pyrolysis, etc.) using organic wastes as raw materials have been investigated with a hope of replacing non-renewable fossil fuels. Among these technologies, the production of microbial lipids through fermentation of oleaginous yeasts coupled with organic wastes consumption is being investigated globally for its potential to simultaneously treat huge amounts of organic waste and produce microbial lipids (i.e., triacylglycerols, TAG) that can be easily converted to fatty acid methyl esters (FAME), namely, a clean renewable fuel, biodiesel [2,3]. Compared to the traditional biodiesel, the microbial-lipids-based biodiesel produced from organic wastes provides several advantages such as lowering production cost, increasing sustainability, and saving land for oil crops [4,5].

Hitherto, microbial fermentation of organic wastes for microbial lipids (i.e., single cell oil) production has been studied using many kinds of oleaginous yeasts (e.g., *Lipomyces starkeyi*, *Rhodotorula glutinis*, and *Rhodosporidium toruloides*) and several organic wastes (e.g., hemicellulosic hydrolysate, sugar beet residue, and rice straw) as feedstock [6,7,8]. As a major class of organic wastes in global megacities, lignocellulosic biomass wastes (LBW) such as orange peel wastes are generated in huge amounts annually and could be a promising carbon source for yeast fermentation [9,10,11]. Among various oleaginous yeasts, *Lipomyces starkeyi* is considered as one of the most promising oil producers, due to its higher oil-producing ability, higher ability to assimilate a broad range of feedstock [12], and ability to tolerate inhibitors in cellulosic hydrolysates [13]. The lipid accumulation in *Lipomyces starkeyi* has been investigated in several aspects such as fermentation mode, strategies for enhancing lipid accumulation [14], transesterification methods [13], lipid metabolism [15], and potential biotechnological applications [16]. To achieve high lipid accumulation, *Lipomyces starkeyi* should be cultivated under several specific conditions, including temperature (e.g., 30 °C) [13], aeration (e.g., 0.25–1 vvm) [17], and pH (e.g., 5–6.5) [18].

During the *Lipomyces starkeyi* growth, the cells tend to proliferate when the carbon, nitrogen and other nutrients are sufficient, while the cells only enter into a lipid accumulation stage when the available nitrogen is sufficiently low [17]. Hence, two-stage fermentation was proposed for *Lipomyces starkeyi* fermentation for a higher lipid accumulation. Indeed, the first-stage fermentation with a relatively low C/N ratio and rich nutrient can allow fast growth and reproduction of yeast cells, while the second-stage fermentation with a relatively high C/N ratio and nitrogen limitation can induce the cells to rapidly accumulate lipids. However, the two-stage fermentation of *Lipomyces starkeyi* has been merely investigated using commercial sugars and chemicals as nutrition sources, resulting in a relatively high operating cost and limitations to subsequent biodiesel production. The development of the biodiesel industry is hampered by the relatively high feedstock costs that covers around 60 to 70% of the total production cost [19]. Fortunately, the low-cost lignocellulosic hydrolysates mainly contain glucose and xylose, which can be used in the two-stage fermentation to partially replace the expensive commercial sugars. Nevertheless, the feasibility of a two-stage fermentation of *Lipomyces starkeyi* grown on sugars derived from LBW for production of microbial lipids and biodiesel remains unclear. 

Therefore, this study aims to fill the aforementioned knowledge gap by investigating two-stage fermentation of *Lipomyces starkeyi* using commercial sugars and sugars derived from LBW, respectively, for the production of microbial lipids and biodiesel. The OD_600_ values, pH values, yeast growth status, C/N ratios, total carbon concentration, total nitrogen concentration, residual sugar concentration, lipid content, lipid titer, and fatty acids profiles of lipids were focused on. The findings of this study would contribute to orange peel waste management and the cost-effective production of microbial lipids and biodiesel, as well as potential environmental benefits.

## 2. Materials and Methods

### 2.1. Feedstock 

Orange peel waste (OP) were obtained from Bengawan Solo Company in Singapore. The OP were dried at 55 °C for 3 days to lower the moisture content. The dried OP was then ground into smaller particles using a blender (Model: AD-G85, X2 Labwares Private Limited, Singapore). Prior to use, the ground OP was sieved using a flatbed sieving machine to obtain the fine particles with diameters <1 mm. Detailed characteristics of the OP used for hydrolysis are presented in Table 1.

### 2.2. Pretreatment of OP for Recovery of Soluble Sugars

To obtain the soluble sugars used as carbon source in yeast fermentation, microwave-assisted alkaline pre-treatment of OP was conducted according to the method reported by Kamalini et al. [20] using a mid-size microwave oven (Panasonic Corporation NN-ST651M, Japan). The microwave pre-treatment was operated at 600 W with 3% (*w/v*) of NaOH solution at an agitation rate of 50 rpm. The reaction time and substrate to liquid ratio were 3 min and 1:62 g/mL, respectively. The microwave-pre-treated OP was cooled down to room temperature for measurement of the concentration of glucose and xylose. Finally, 4 M of HCl solution was used to adjust the pH of the hydrolysate to 6.0. 

### 2.3. Yeast Strain and Medium

The oleaginous yeast *Lipomyces starkeyi* (NRRL Y-1388) was purchased from the Bio-REV PTE LTD and then stored at −80 °C. The yeast extract peptone dextrose (YPD) liquid medium (pH = 6) contained 10 g/L yeast extract, 20 g/L peptone, and 20 g/L glucose. The YPD liquid medium was used as the one-stage fermentation medium (pH = 6.0). All the commercial chemicals were of analytical reagent grade and purchased from the Sigma-Aldrich Pte Ltd. All the media were sterilized by autoclaving at 121 °C for 20 min before the cultivation experiment. To prepare seed culture, *Lipomyces starkeyi* was pre-cultured in 250 mL flasks containing 50 mL YPD medium for 4 days to reach logarithmic phase. 

### 2.4. One-Stage and Two-Stage Fermentation of Yeast Using Commercial Sugars (YPD Medium) and OP Hydrolysate

The pre-cultured *Lipomyces starkeyi* cells were then transferred into the 250 mL flasks (50 mL in working volume) for one- and two-stage fermentation at 30 °C and 200 rpm for 7 d (Table 2). Initial pH values for both one-stage and two-stage fermentation were adjusted to around 6.0 by using 4 M HCl solution and 4 M NaOH solution. 

Regarding the two-stage fermentation of yeast using commercial sugars, YPD medium was used in the first-stage bioreactor to cultivate the yeast for 5 d (Table 2). The time duration of 5 d for the first-stage fermentation was selected based on a previous study on the yeast cell concentration at different fermentation periods by Yamazaki et al. [21], who found that the cell concentration of *Lipomyces starkeyi* reached the highest point at day 5. Subsequently, yeast cells were harvested through centrifugation at 8000 g for 5 min and were washed twice with sterile distilled water before being re-suspended in the second-stage bioreactor. The medium for the second-stage fermentation was 60 g/L glucose solution according to a previous study [22]. Regarding the one-stage fermentation using bio-wastes-derived sugars, OP hydrolysate was supplemented with a certain amount of xylose and glucose to achieve identical xylose and glucose concentration to the YPD medium in Section 2.3. Regarding two-stage fermentation of bio-wastes-derived sugars, the first stage used the modified OP hydrolysate for 5 days of fermentation, while the second stage used 60 g/L glucose solution (Table 2). Other experimental conditions of one-stage and two-stage fermentation using bio-wastes-derived sugars were identical to the fermentation with YPD medium. Initial pH was adjusted to around 6.0 by 4 M HCl solution and 4 M NaOH solution. To guarantee the repeatability, all the experiments were carried out in triplicate under the same experimental conditions. During the fermentation processes, 15 mL of fermentation broth was sampled once per two days from each flask to measure the parameters including optical density at 600 nm (OD_600_), pH, microscopic examination, TC, TN, sugar concentration, lipid titer, and lipid content. At the end of the fermentation, all the yeast cells were harvested by centrifugation of the collected fermentation broth. The collected yeast cells were washed twice and then dried using a freeze dryer for 24 h. The dried cells were used for lipid extraction. 

### 2.5. Extraction of Microbial Lipids and Production of Biodiesel

The total lipids were extracted using a modified Bligh & Dyer method [23]. Briefly, 400 mg of dried yeast cells were weighed and digested with 10 mL of 4 M HCl at 60 °C for 1 h to achieve cell membrane lysis. Afterward, 15 mL of 1:1 (*v/v*) chloroform: methanol mixture was used to extract lipids for 1 h. The mixture was then centrifuged using a high speed refrigerated centrifuge (Model 6000, KUBOTA Corporation, Osaka, Japan) at 5000 rpm for 10 min, followed by transferring the organic phase to a new container. For the residue, a second extraction was performed. Subsequently, the chloroform layers in the extracts were evaporated at 40 °C for 24 h. To convert lipids to FAME, 70 mg lipid was weighed and transferred to a 100 mL round-bottomed flask, followed by adding 15 mL of 5% (*w/v*) NaOH in methanol. The round-bottomed flask was placed in a fume hood to carry out the transesterification process at 65 °C for 50 min. After cooling down, 2 mL n-Hexane was added to the mixture, vortexed thoroughly, and centrifuged to separate the organic layer. 

### 2.6. Analytical Methods

Standard curves of measuring concentration of glucose and xylose were established according to the dual-wavelength spectroscopic method reported by Chi et al. [24]. The calculation Equations (1–3) of standard curves for total sugar concentration, xylose concentration, and glucose concentration were obtained below.

(1) $$C_{T}\left(\frac{mmol}{L}\right)=113.636*A_{425}$$

(2) $$C_{xylose}\left(\frac{mmol}{L}\right)=4.272*A_{553}$$

(3) $$C_{glucose}\left(\frac{mmol}{L}\right)=113.636*A_{425}-4.272*A_{553}$$

where *C_T_*, *C_xylose_*, and *C_glucose_* were the total sugar concentration, *xylose* concentration, and glucose concentration, respectively. *A*_425_ and *A*_553_ were absorbance values at 425 nm and 553 nm, respectively. 

Total biomass concentration (g/L) was defined through the Equation (4):

(4) $$\mathrm{Total}\;\mathrm{biomass}\;\mathrm{concentration}=\frac{Dry\;cell\;weight\;\left(g\right)}{Culture\;medium\;volume\;\left(L\right)}$$

To obtain the dry cell weight, 5 mL culture suspension was transferred to a pre-weighed pellet and centrifuged at the speed of 10,000 rpm for 8 min. The pellet was washed with distilled water to remove the impurities (e.g., salts), and was then dried until constant weight at 60 °C for weighing dry cell weight. 

Lipid titer (g/L) was defined and calculated via the following Equation (5):

(5) $$\mathrm{Lipid}\;\mathrm{titer}=\frac{Lipid\;weight\;\left(g\right)}{Culture\;medium\;volume\;\left(L\right)}$$

To obtain the lipid weight, the intracellular lipid was extracted by the previously reported lipid extraction method [25,26,27]. 

Lipid content (%) was calculated according to the Equation (6):

(6) $$\mathrm{Lipid}\;\mathrm{content}=\frac{Lipid\;weight\;\left(g\right)}{Dry\;cell\;weight\;\left(g\right)}$$

pH and elemental compositions were determined according to the corresponding methods described by Zhang et al. [28]. VS and TS of the samples were determined via the weighing method described by Lin et al. [29]. The cellulose and hemicellulose contents were determined using the method reported by Van Soest et al. [30]. The obtained FAME was filtered twice using the 0.45 μm filters and then transferred into sample vials for the analysis of the fatty acid compositions in FAME using a GC system (Clarus 580 GC, PerkinElmer, Waltham, MA, USA). All the obtained peaks were subjected to an analysis against the standard curves for peak identification and quantitation. 

### 2.7. Statistical Analysis

A computer-assisted statistics program, the SAS System for Windows (version 6.1, SAS Institute Inc., Cary, NC, USA), was utilized to analyse the variance (ANOVA) of the parameters (e.g., fatty acid percentages) among different treatments, with a significance level of 5% (*p*-value < 0.05).

## 3. Results and Discussion

### 3.1. OD_600_, pH, and Microscopic Images of the Yeast Cells during Fermentation

**Figure 1:**
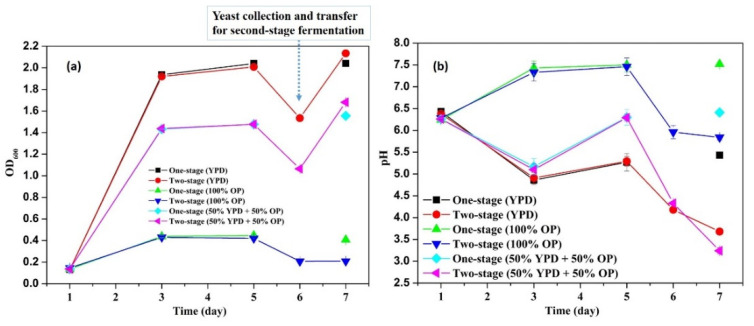
OD_600_ values (**a**) and pH values (**b**) during fermentation with various media. For two-stage fermentation, the yeast cells were collected and re-suspended in a mixture solution of xylose and glucose at day 6.

The values of OD_600_ can represent the dynamic growth rates of the microbial cells [31,32]. From Figure 1a, it was found that the values of OD_600_ at day 1 during fermentation with various media were appropriately 0.14 ± 0.01. After two days, the values of OD_600_ increased to 1.93 ± 0.02, 0.43 ± 0.01, 1.44 ± 0.01 in flasks with YPD, 100% OP, and 50% YPD + 50% OP, respectively. The results indicated that the YPD medium was more beneficial than 50% YPD + 50% OP for the growth of *Lipomyces starkeyi*, followed by pure OP (i.e., orange peel waste hydrolysate). The slowest growth rate in the medium of pure OP might be ascribed to the relatively high pH value (i.e., 7.43 ± 0.16, Figure 1b), which was beyond the appropriate pH range of 3 to 6.5 [17,18]. From the 3rd to 5th day, the flasks with YPD and 50% YPD + 50% OP showed a slight increase in OD_600_, while the flasks with pure OP kept a relatively stable OD_600_. The results indicated that the yeast cell growth from the 3rd to 5th day was extremely slow. The yeast cells in the two-stage fermentation groups were collected at day 6 and then re-suspended in the second-stage flasks with pure sugar solution. 

**Figure 2:**
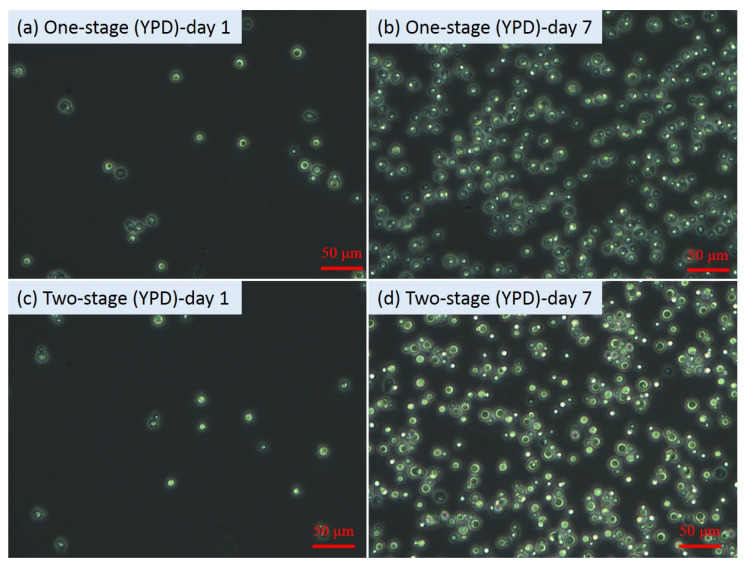
Microscopic images of yeast cells during fermentation with YPD.

**Figure 3:**
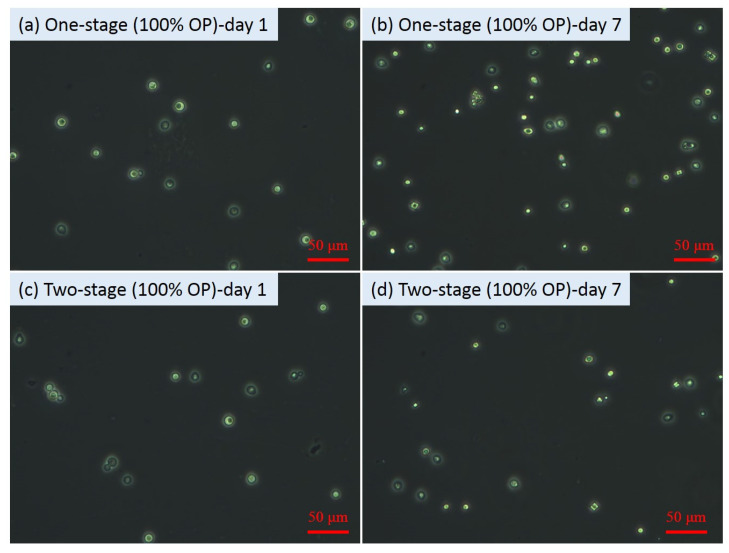
Microscopic images of yeast cells during fermentation with 100% orange peel hydrolysate.

**Figure 4:**
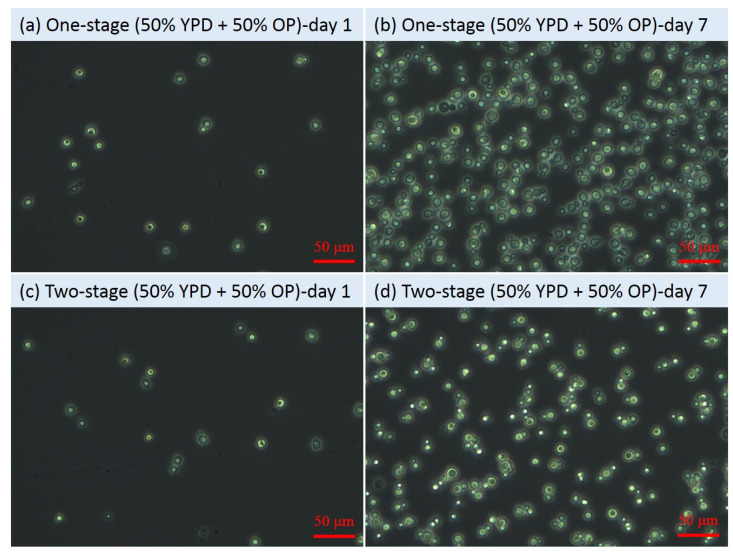
Microscopic images of yeast cells during fermentation with 50% YPD and 50% orange peel hydrolysate.

From Figure 1a, after re-suspension at day 6, the OD_600_ values of all the second-stage fermentation flasks decreased compared to those before re-suspension, which might be mainly attributed to the lighter colour of the sugar solution than the original fermentation media. However, after one day of second-stage fermentation, the OD_600_ values increased significantly (*p* values < 0.05) from 1.53 ± 0.01 to 2.13 ± 0.02 for the flasks with YPD and from 1.06 ± 0.00 to 1.68 ± 0.01 for the flasks with 50% YPD + 50% OP. Nevertheless, no obvious increase in the OD_600_ value (i.e., 0.21) in the flasks with pure OP hydrolysate was observed. The cell growth status reflected by the OD_600_ results were consistent with the results shown in the microscopic images. At the end of the fermentation (i.e., 7th day), the microscopic images (Figure 2, Figure 3 and Figure 4) of the yeast cells in different flasks were obtained. It was found from the microscopic images that the yeast cells grew normally in the flasks with YPD or 50% YPD + 50% OP medium, showing round or oval shapes; however, the yeasts cells in pure OP hydrolysate grew abnormally, showing skinny and angular shapes. These results demonstrated that the second-stage fermentation of *Lipomyces starkeyi* was effective in medium YPD and 50% YPD + 50% OP, but ineffective in medium of pure OP hydrolysate. On the one hand, the poor cell growth in medium of the pure OP hydrolysate could be attributed to the lack of necessary trace elements (e.g., Fe, Zn, Mn, Co and Cu) compared to the YPD medium. To tackle this issue, bioaugmentation with essential trace nutrition could be a promising method to enhance the fermentation process using OP hydrolysate as feeding [33,34]. On the other hand, the microwave-assisted alkaline pre-treatment of OP could produce some inhibitory substances such as D-limonene, which could inhibit the fermentation process [35]. 

Furthermore, from Figure 2b,d, the internal lipid droplet in two-stage fermentation with YPD was bigger than that in one-stage fermentation. A similar phenomenon was also observed in two-stage fermentation with 50% YPD + 50% OP. However, the two-stage fermentation with pure OP hydrolysate failed to show the similar enhancing effects. The results showed that an additional second-stage fermentation contributed to the lipid accumulation in the yeast cells with normal growth conditions. Previously, Liu et al. [22] reported that the two-stage fermentation method could be a promising method to enhance lipid production from lignocellulose hydrolysates. The present study not only confirmed the advantage of the two-stage fermentation in enhancing lipid accumulation, but further showed that the advantage relied on the normal growth of yeast cells as a prerequisite. 

### 3.2. C/N Ratio, TC Concentration, TN Concentration, Residual Sugar Concentration during Fermentation

**Figure 5:**
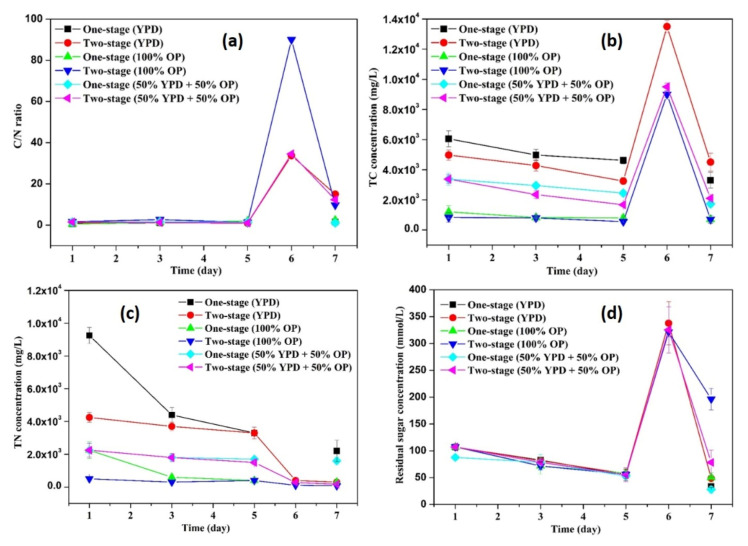
Dynamic changes of C/N ratio (**a**), TC concentration (**b**), TN concentration (**c**), residual sugar concentration (**d**) during fermentation.

To investigate the effects of carbon/nitrogen source and sugar concentration on yeast cell growth and lipid accumulation in *Lipomyces starkeyi*, the C/N ratio, TC concentration, TN concentration, and residual sugar concentration in each flask were monitored. Figure 5a displays the changes of the C/N ratios along with production of yeast cells and lipids. From day 1 to day 5, no significant difference (*p* values > 0.05) in the C/N ratios among different flasks was observed. The relatively stable C/N ratios indicated that the carbon source and the nitrogen source were consumed in a relatively consistent pace between day 1 and day 5. The dynamic changes of TC and TN concentrations shown in Figure 5b,c also supported the above-mentioned results. In the meantime, the sugar concentration decreased from 103.83 ± 7.24 to 55.79 ± 1.68 mmol/L due to consumption by cells. After cell collection and re-suspension, the second-stage flasks showed the higher C/N ratios (Figure 5a), which was attributed to the nitrogen limitation of new sugar solution in the second-stage flasks (Figure 5b–d). After one day fermentation, the carbon source was significantly (*p* values < 0.05) consumed by the yeasts for lipid accumulation at the second-stage flasks, which was consistent with the relatively larger internal lipid droplets (Figure 2d and Figure 4d). It has been found that, yeast cells only entered a lipid accumulation phase when the available nitrogen concentration was sufficiently low [36]. By harvesting the cells at day 6 and re-suspending the cells in a nitrogen-limited sugar solution, the cells entered a lipid accumulation phase by consuming extra sugar, leading to a higher lipid content (see Section 3.3). Moreover, two-stage flasks with different media showed diversified C/N ratios, TC concentration, and TN concentration, but with similar changing tendency. The results further demonstrated that the two-stage fermentation method was of great potential in lipid production by *Lipomyces starkeyi*. 

### 3.3. Lipid Content and Lipid Titer during Fermentation

**Figure 6:**
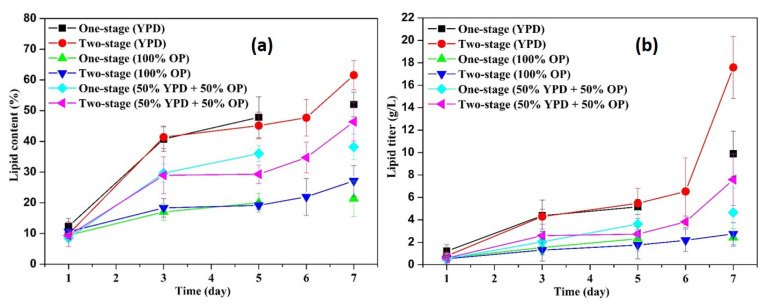
Lipid content (**a**) and lipid titer (**b**) during fermentation.

Figure 6 shows the lipid content and lipid titer in different flasks. In general, the lipid contents and lipid titer in all the flasks showed a continuously increasing tendency, indicating that the cell growth and lipid biosynthesis kept active throughout the entire fermentation process. Specifically, at day 1, the lipid contents in flasks with YPD, 100% OP, and 50% YPD + 50% OP were 9.8–12.3, 9.4–10.5, 8.5–9.3, respectively. After 4 days of fermentation, these values increased to 45.13–47.8, 19.1–20.3, and 29.3–30.0, respectively. Furthermore, these values increased to 52.0–61.6, 21.34–27.13, and 38.18–46.35, respectively, at the end of the fermentation. More specifically, at day 7, the lipid contents in two-stage flasks with YPD, 100% OP, and 50% YPD + 50% OP were 18.5%, 27.1%, and 21.4% higher than those of the corresponding one-stage fermentation. The relatively higher lipid contents in two-stage fermentation were consistent with the relatively larger internal lipid droplets of the yeast cells in the second-stage flasks (Figure 2d and Figure 4d). It has been reported that the adaptation of the yeast to the hemicellulosic hydrolysate contributed to better fermentation performance with no lag phase [37]. Hence, in order to enhance the yeast fermentation using OP medium, the adaptation approach deserves to be incorporated into the two-stage fermentation in future studies.

Regarding the lipid titer, from Figure 6b, it was found that the harvested lipids from the one-stage fermentation flasks were 9.89 g/L for YPD medium, 2.42 g/L for OP medium, and 4.65 g/L for the 50% YPD + 50% OP medium, respectively. After the two-stage fermentation, the corresponding values increased to 17.58 g/L, 2.75 g/L, and 7.58 g/L, respectively. In contrast, the two-stage fermentation enhanced the lipid titer by 77.8%, 13.6%, and 63.0% for YPD medium, OP medium, and the 50% YPD + 50% OP medium, respectively. The increased lipid titer in two-stage fermentation can be mainly ascribed to the enhanced microbial growth (i.e., OD_600_ in Figure 1a) and the enhanced lipid content (Figure 6a). Therefore, in order to achieve the highest lipid production, yeast cells can be firstly cultivated in a nutrient-rich medium to achieve the highest cell numbers. Afterward, the substantial number of cells are harvested and transferred into a nitrogen-limited sugar solution to further storage lipid. In addition, it can be concluded from the diversified values in Figure 6 that the lipid contents and lipid titer can be greatly affected by the fermentation media. This finding is also supported by previous studies. For instance, Matsakas et al. [38] obtained a lipid content of 29.5% (*w/w*) using juice from saccharified sweet sorghum stalks as feedstock. Di Fidio et al. [39] successfully converted paper mill waste to single cell oil by the yeast *Lipomyces starkeyi* with a lipid content of 37 wt% and a lipid titer of 3.7 g/L. A lipid content of 21.3% was achieved by Islam et al. [40], who adopted palm oil mill effluent as feedstock for *Lipomyces starkeyi* for lipid production. Huang et al. [41] used corncob acid hydrolysate for microbial oil production by the yeast *Lipomyces starkeyi* and achieved a lipid content of 47% and a lipid yield of 8.1 g/L. Overall, *Lipomyces starkeyi* is a promising strain for microbial oil production using sugars derived from organic wastes; however, the fermentation process needs continuous efforts to optimize before achieving the maximized lipid production.

### 3.4. Fatty Acids Profile of the Lipid Samples of Lipomyces starkeyi after Fermentation

The fatty acids profiles of the lipid samples of *Lipomyces starkeyi* in different fermentation processes were determined. The results showed that the fatty acid compositions were mainly dominated by two fatty acids, namely, palmitic acid (C16:0) and oleic acid (C18:1). The relative contents of palmitic acid and oleic acid in various flasks were approximately 33.36–38.43% and 46.6–48.12%, respectively. The remaining fatty acids included stearic acid (C18:0) with a content of 4.59–5.97%, palmitoleic acid (C16:1) with a content of 3.01–3.96%, and linoleic acid (C18:2) with a content of 1.12–2.93%, respectively. Such a fatty acids profile, similar to the main components of vegetable oil, was consistent with several previous studies [42,43,44]. The differences in fatty acids profiles among different fermentation flasks were analysed by significance analysis. The results showed that there was no significant difference (*p* values > 0.05) between any two flasks, which demonstrated that the environmental factors had limited influence on the fatty acids profiles in microbial lipids of the yeast *Lipomyces starkeyi.*

To go a step further, several studies can be performed based on the findings of this study. First, the supplementation of trace elements into OP hydrolysate prior to use should have a great potential to contribute to the two-stage fermentation of yeast *Lipomyces starkeyi* using non-expensive feedstock for microbial lipid production. Second, the large-scale test-bedding of the two-stage fermentation using *Lipomyces starkeyi* should be explored to provide pivotal technical supports for potential pilot- and commercial-scale biorefinery plants. Thirdly, the integration between the adaptive laboratory evolution approach and rational genetic engineering of the *Lipomyces starkeyi* strain holds great potential to obtain the artificially evolved yeast cells and genetically engineered yeast strains, which could utilize wider carbon sources and tolerant more inhibitors, leading to a better performance on cell growth and lipid accumulation. 

## 4. Conclusions

The excellent ability of lipid accumulation of the oleaginous yeast *Lipomyces starkeyi* has been well demonstrated. The present study aims to reduce the operating cost by exploring the feasibility of a two-stage fermentation of *Lipomyces starkeyi* grown on YPD, orange peel hydrolysate medium, and mixture medium of YPD and orange peel hydrolysate for production of microbial lipids and biodiesel. In the fermentation process for seven days, microscopic image analyses confirmed the advantage of the two-stage fermentation with YPD and 50% YPD + 50% OP medium in accumulating larger internal lipid droplets in the yeast cells. In contrast, the growth status of the yeasts cells in pure OP hydrolysate was not satisfied, which might be due to the lack of certain trace elements and the existence of some inhibitors in OP hydrolysate. Compared to one-stage fermentation, the lipid contents were increased by 18.5–27.1% and the lipid titer was enhanced by 13.6–77.8% by a two-stage fermentation. The microbial lipids obtained from both one-stage and two-stage fermentation were dominated by two fatty acids, namely, palmitic acid (C16:0, 33.36–38.43%) and oleic acid (C18:1, 46.6–48.12%) and showed similar fatty acids components to vegetable oil. The results from this study not only confirmed the significance of nutrition formulation in yeast fermentation media, but also provided a two-stage fermentation approach as a promising way to facilitate lipid accumulation in the oleaginous yeasts. The findings of this study would be beneficial to economically manufacture biodiesel by using microbial lipids as a feedstock and to resource recovery of orange peel waste, which would jointly contribute to the environmental protection. Next, to help realize the industrialization of microbial lipids from yeast *Lipomyces starkeyi*, the supplementation of trace elements into OP hydrolysate, the large-scale test-bedding of the two-stage fermentation, and the incorporation of the adaptive laboratory evolution approach and rational genetic engineering can be investigated.

## Figures and Tables

**Table 1:** Detailed characteristics of the orange peel used for hydrolysis.

Characteristics	Unit	Orange Peel
Extractives ^1^	wt% ^2^	88.76 ± 4.76
Cellulose	wt% ^2^	2.98 ± 0.68
Hemicellulose	wt% ^2^	5.79 ± 1.11
Lignin	wt% ^2^	0.47 ± 0.10
Volatile solids (VS)	wt% ^3^	18.01 ± 0.37
Total solids (TS)	wt% ^3^	23.96 ± 0.15
VS/TS ratio	-	0.752
Carbon	wt% ^2^	41.14 ± 1.73
Hydrogen	wt% ^2^	5.63 ± 0.19
Nitrogen	wt% ^2^	1.00 ± 0.04
C/N ratio	-	41.14

^1^ Extractives represent organic compounds (e.g., protein, carbohydrate, and lipid, etc.) extracted from the sample using the neutral detergent (3% SDS). ^2^ On dry basis. ^3^ On wet basis.

**Table 2:** Experimental design of one-stage and two-stage fermentation.

Flask Label	Fermentation Type	Fermentation Medium and Time	
		First-Stage	Second-Stage
One-stage (YPD)	One-stage fermentation of commercial sugars	YPD medium; 7 d; Cell collection	Not applicable
Two-stage (YPD)	Two-stage fermentation of commercial sugars	YPD medium; 5 d; Cell collection; Transfer to the second stage	60 g/L glucose; 1 d
One-stage (100% OP)	One-stage fermentation of bio-wastes-derived sugars	Modified bio-wastes-derived OP hydrolysate (with identical xylose and glucose concentration to YPD medium) ^1^; 7 d; Cell collection	Not applicable
Two-stage (100% OP)	Two-stage fermentation of bio-wastes-derived sugars	Modified bio-wastes-derived OP hydrolysate (with identical xylose and glucose concentration to YPD medium); 5 d; Cell collection; Transfer to the second stage	60 g/L glucose; 1 d
One-stage (50% YPD + 50% OP)	One-stage fermentation of commercial sugars and bio-wastes-derived sugars	50% YPD + 50% OP; 7 d; Cell collection	Not applicable
Two-stage (50% YPD + 50% OP)	Two-stage fermentation of commercial sugars and bio-wastes-derived sugars	50% YPD + 50% OP; 5 d; Cell collection; Transfer to the second stage	60 g/L glucose; 1 d

^1^ The modified bio-wastes-derived OP hydrolysate was supplemented with certain amount of xylose and glucose to achieve the identical sugar concentration to YPD medium.

## Data Availability

Not Applicable.
